# Based on Plasma Metabonomics and Network Pharmacology Exploring the Therapeutic Mechanism of *Gynura procumbens* on Type 2 Diabetes

**DOI:** 10.3389/fphar.2021.674379

**Published:** 2021-05-28

**Authors:** Wenjing Guo, Hui Ouyang, Mi Liu, Jiahui Wu, Xiao He, Shilin Yang, Mingzhen He, Yulin Feng

**Affiliations:** ^1^ Jiangxi University of Chinese Medicine, Nanchang, China; ^2^ National Pharmaceutical Engineering Center for Solid Preparation in Chinese Herbal Medicine, Nanchang, China

**Keywords:** *Gynura procumbens*, type 2 diabetic, metabonomics, network pharmacology, Western blot

## Abstract

*Gynura procumbens* (GP) is a perennial herbal medicine and food homologous plant, which has been reported to have a good hypoglycemic effect. However, its active components and underlying mechanism of action are not clear. Here, we aimed to confirm the effects of GP on type 2 diabetes (T2DM) from several different aspects. We used UPLC/Q-TOF MS to analyze the metabolic patterns, which included blood samples of clinical subjects and *db*/*db* mice to screen for serum metabolic markers and metabolic pathways. We also used network pharmacology to study GP targets in the treatment of T2DM. Data from endogenous metabolites in plasma showed that two common pathways, including glycerol phosphate metabolism and retinol metabolism, were identified in plasma samples of the groups. Finally, Western blot analysis was used to verify the expression of proteins in the PI3K/AKT and AGE–RAGE signaling pathways. The protein expression of AKT, eNOS, iNS, and MAPK was significantly upregulated, and the expression of caspase-8 and caspase-3 was significantly downregulated. Thus, our findings indicated that GP could alleviate insulin resistance by regulating biometabolic markers and key proteins in the PI3K/AKT and AGE–RAGE signaling pathways to treat T2DM.

## Introduction

Type 2 diabetes mellitus (T2DM) is a chronic metabolic disease with clinical symptoms, such as chronic hyperglycemia and insulin resistance. Uncontrolled diabetes can lead to long-term health complications, including blindness, stroke, kidney failure, and heart disease (Veronese et al., 2019). Referred to the latest data from the International Diabetes Federation, a significant number of individuals with diabetes aged 20–79 years have reached 425 million worldwide, of which T2DM accounts for 90–95% (Roden and Shulman, 2019; Zimmet et al., 2014). It has been estimated that by the year 2045, there will be 629 million diabetic patients. At present, oral hypoglycemic drugs and insulin injections are the main strategies that are used for the treatment of diabetes. Although sulfonylureas and biguanides can control the blood glucose level of patients, these drugs have shown adverse side effects in the clinic and basically have no protective effect on islet B cells (Chen et al., 2017). Therefore, an increased number of individuals prefer to use natural products to prevent and treat T2DM (Cortés-Martín et al., 2021).

GP is a perennial herbal medicine and a food homologous plant, which is a type of green vegetable. It has previously been reported that GP has a good hypoglycemic effect, which has accumulated rich experience in the treatment of T2DM; however, its active components and underlying mechanism of action are not clear (Guodong et al., 2013). In the present research, we hypothesized that GP extracts help improve glucose tolerance and lower the blood sugar level to a normal level and has minimal side effects. The effect of GP is better than that of sulfonylureas and sulfonylureas which are chemical drugs (Tan et al., 2016). The hypoglycemic mechanism of GP involves repairing injured islet cells and stimulating the normal secretion of hormones, thereby inhibiting the level of α-glycosidase, which slows down the production and absorption of glucose and increases the synthesis of muscle glycogen in the body (Hassan et al., 2010). The main hypoglycemic machinery of GP is likely to increase the ability to scavenge free radicals and inhibit the damage to islet cells, which restores the secretory function of the islet cells to a normal level and finally plays a role in lowering blood sugar levels (Li et al., 2020). However, whether GP has other antidiabetic mechanisms remains to be further studied.

Metabonomics, a technique for the analysis of metabolites and pathways *in vivo* (Cortés-Martín et al., 2021), provides comprehensive and reliable information for the treatment of T2DM. In addition, network pharmacology technology, a virtual computer contract method, has been applied in the research of active ingredients and potential targets of traditional Chinese medicine successfully. In this study, we focused on plasma metabolomics, network pharmacology technology, and Western blot analysis to evaluate the antidiabetic effects of GP on mice with T2DM. We aimed to identify common metabolic pathways in T2DM mice and human T2DM by Western blot analysis to evaluate the expression of pivotal proteins that are involved in T2DM. The underlying pharmacological mechanisms of GP have been revealed and are beneficial for the clinical application of GP in the treatment of diabetes.

## Materials and Methods

### Materials

GP was collected from Jing’an County, Jiangxi Province, and identified as *Gynura procumbens* (Lour.) Merr whole grass provided by Professor Guoyue Zhong of the Jiangxi University of Chinese Medicine was deposited in the Center of National Medicine Resource, and Voucher specimens (accession number JX-20170826-A) were deposited in the Center of National Medicine Resource, Jiangxi University of Chinese Medicine, Nanchang, China. As a control, 2-chloro-l-phenylalanine was used, which was purchased from Shanghai McLean Biochemical Technology Co., Ltd. (Shanghai, China). Acetonitrile and methanol (HPLC grade) were purchased from Fisher Scientific (Waltham, MA, United States). Formic acid (analysis grade) was purchased from China Chemical Reagent Co., Ltd. Primary antibodies directed against GAPDH (Abcam, United Kingdom), AKT (Abcam, United Kingdom), eNOS (Abcam, United Kingdom), iNS (Abcam, United Kingdom), MAPK (Abcam, United Kingdom), caspase-8 (Abcam, United Kingdom), and caspase-3 (Abcam, United Kingdom), and lgG secondary antibody were purchased from Cell Signaling Technology (Beverly, MA, United States).

### Source of Patients With Abnormal Blood Glucose

The clinical samples of the study came from the Department of Physical Examination of the affiliated Hospital of Jiangxi University of Chinese Medicine (Nanchang, China). The samples were collected from September to the end of November 2018, and the study followed the guidelines of the Ethics Committee of the affiliated Hospital of Jiangxi University of Chinese Medicine (JXFYLL2017103013). All volunteers were aware of the entire process and details of the experiment and signed informed consent. In total, 30 control cases and 30 patients with an abnormal blood glucose level were selected.

### Animals

Experimental animals were purchased from Changzhou Cavens Experimental Animal Co., Ltd. (license No.: SCXK (Su) 2016-0010) (Changzhou, China). Mice included 30 C57BL/KsJ-*db*/*db* mice aged 6 to 8 weeks weighing between 20 and 22 g (*db*/*db* mice were derived from autosomal recessive inheritance of an C57BL/KsJ inbred strain and were used as the T2DM model), and another 10 were male wild-type mice of the same age. The certificate number of this batch of animals was No. 201822505. All animals were of specific pathogen-free (SPF) grade and were kept in the animal room in the SPF environment of our facility. The animal room was kept on a schedule with alternating light and dark cycles for 12 h, and all mice were free to drink and eat. The chow was synergistic irradiation breeding feed. The constant temperature of the feeding environment was 25 ± 1°C, the relative humidity was 55–65%, and the indoor air circulation was maintained.

### Study Design

A total of 1,750 g of fresh dried GP was weighed, refluxed, and extracted with 5,000 ml of 70% ethanol for 1.5 h. It was repeat-operated two times, and extracts were combined, decompressed, and concentrated to dry. The dried extract was 241.32 g, and the extraction rate of GP was 16.07%. GP was added to normal saline before use, and the extract was fully dissolved and suspended by ultrasound and was configured into a suspension of the required concentration. After comprehensive investigation of the literature and previous experiments, the gastric perfusion dose of *db*/*db* diabetic mice was set to 3 g/kg. The positive drug used in this study was metformin (MET) hydrochloride tablets with a specification of 0.5 g/tablets. To maintain consistency, MET tablets were placed into a 50-ml sterilized Eppendorf tube and normal saline was added and ultrasound treatment was performed to fully dissolve and configure a required concentration of MET solution. Based on the clinical dose and relevant literature reports, a dose of 0.2 g/kg MET was chosen in this study.

### Dosage Information

After 1 week of adaptive feeding, animals were divided into four groups with 10 mice in each group randomly. Mice in the control and model groups received intragastric administration of normal saline, mice in the MET group received MET hydrochloride tablets, 0.2 g/kg, and mice in the GP group received GP extract, 3 g/kg. The volume of administration was 8 ml/kg.

### Sample Preparation

After 5 weeks of administration, mice were fasted of water after the last administration, orbital blood samples were obtained, and mice were sacrificed. The blood samples were placed at room temperature for 2 h and centrifuged in low temperature and highly speed centrifuge for 15 min (4°C, 4,000 rpm). The supernatants were transferred to a sterile EP tube and stored at −80°C for serological testing. Tissue samples were placed on ice, the abdomen was cut open, and the pancreas was separated between the duodenum and the spleen, then the tail of the pancreas near the spleen was quickly removed for subsequent analysis.

### Metabolomics Analysis

#### Sample Pretreatment

Metabolomics was performed using the plasma of 30 patients in the clinical diabetes mellitus group and mouse plasma samples from mice in the control, model, MET, and GP groups. Plasma supernatants were treated with acetonitrile containing 2-chloro-l-phenylalanine (10 μg/ml) at a ratio of 5:1 (acetonitrile: plasma supernatant, V/V, 200 μl). Then the samples were centrifuged (1,200 g, 15 min) at 4°C, and 10 μl of all the centrifuged supernatants, which were evenly mixed, served as the quality control (QC) sample. Last, samples were analyzed by LC-MS (Wang et al., 2019).

#### LC-MS Analysis

A UHPLC (ESI) system (Shimadzu, Kyoto, Japan) and an AB Sciex quadrupole time-of-flight mass spectrometer (TripleTOF^®^ 5600, AB SCIEX, Framingham, MA, United States) were used for LC-MS analysis. A Waters ACQUITY UHPLC BEH C18 column (100 mm × 2.1 mm, 1.7 μm, water) was used at a flow rate of 0.3 ml/min, and the injection volume was 3 μl. The mobile phase consisted of 0.1% formic acid (A) and acetonitrile (B), and the gradient of the mobile phase B was as follows: 0.01–3 min, 10–20% B; 3–5 min, 20–40% B; 5–7 min, 40–60% B; 7–9 min, 60–80% B; 9–11 min, 80–90% B; 11–15 min, 90–95% B; 15–20 min, 95% B; 20–22 min, 95-5% B; and 22–25 min, 5% B. TOF-MS and TOF-MS/MS were performed simultaneously. The range of TOF-MS was from 50 to 1,250. When the collision reached 40 eV, the eight most intense ions from each TOF-MS scan were selected for TOF-MS/MS. To ensure data quality, previously described approaches were adopted (Huang et al., 2013; Zhang et al., 2018; Favari et al., 2020). Moreover, to maintain data accuracy and stability, the TOF-MS was calibrated after every five samples. Simultaneous LC-MS analysis was performed for QC (*n* = 6) and plasma samples. From QC samples, the relative standard deviations (RSDs) of the retention times and typical peak intensities (including internal standards) were used for the evaluation of data quality.

#### Data Analysis

As mentioned before, data were analyzed according to a previously described method (Theodoridis et al., 2012). First, Marker View 1.2.1 software was used to convert the raw LC-MS data into the “M/Z” data file format. Before chemometric analysis, the date obtained for each sample was normalized by comparison with the internal standard (2-chloro-l-phenylalanine). The detection frequencies (DFs) and relative standard deviations (RSDs) of each group were used for data screening. These characteristics were statistically analyzed only when the DFs of either group reached 100% and the RSD was less than 30%. Missing values were replaced by semi-minimum values with rich features. To guarantee the reliability of the data quality and model, we used the principal component analysis (PCA) and orthogonal projection discriminant analysis (OPLS-DA) potential energy structure. Transcriptomic and metabolomic profiling reveals the protective effect of *Acanthopanax senticosus* (Chen et al., 2021). To avoid excessive OPLS-DA model overfitting, we used Simca-P software to the default 200 random seven cross validation and test. Choosing the OPLS-DA model of VIP scores > 1 and *p* values < 0.05 features and the human metabolome database (HMDB), the candidate metabolites were identified. Relevant metabolic pathways were established by integrating the small-molecule pathway database (SMPDB)/Kyoto Encyclopedia of Genes and Genomes (KEGG) with Metobanalyst 4.0 (Kanehisa et al., 2000; Jewison et al., 2014; Jasmine and Jianguo, 2018). For reference compounds, metabolites with a significant effect on metabolic pathways that cause significant changes in plasma in the clinical diabetic group and model mice were identified as potential biomarkers (Bonny et al., 2011). Data were analyzed by Student’s *t* test. *p* < 0.05 was considered statistically significant.

### Network Pharmacology Research

All the bioactive GP compounds were collected from the Traditional Chinese Medicine Systems Pharmacology Database and Analysis Platform (TCMSP, https://tcmspw.com/tcmsp.php), (Ru et al., 2014), Integrative Pharmacology–based Research Platform of Traditional Chinese Medicine (TCMIP, http://www.tcmip.cn/TCMIP/index.php/Home/Index/index.htm) (HY et al., 2019), DrugBank (https://go.drugbank.com/) (Wishart et al., 2017), and SwissTargetPrediction (http://www.swisstargetprediction.ch/). The components selected met the following criteria: oral bioavailability (OB) ≥3 0%, drug-likeness (DL) ≥ 0.18, or drug-likeness weight (TCMIP) ≥ 0.40 so as to identify as many fully active compounds as possible. Components were used to construct a “compound–compound target network” of GP through Cytoscape 3.7.2 (Shannon et al., 2003). The terms “type II diabetes mellitus” and “T2DM” were used as the keywords to retrieve disease-related genes from the Online Mendelian Inheritance in the Man^®^ (OMIM) database (OMIM; https://omim.org/), GeneCards database (http://www.genecards.org) (Stelzer et al., 2016), Therapeutic Target Database (TTD, http://db.idrblab.net/ttd/) (Wang et al., 2020), and the Encyclopedia of Traditional Chinese Medicine (ETCM, http://www.tcmip.cn/ETCM/index.php/Home/Index/index.html) (Xu et al., 2019).

Venn analysis was performed through an online Web site (Bioinformatics, http://bioinformatics.psb.ugent.be/webtools/Venn/) (Kanehisa et al., 2004) to overlap GP-related targets and selected targets of GP to further clarify the potential mechanism of GP in T2DM treatment. Based on the intersecting network, the plug-in “CytoHubba” (http://apps.cytoscape.org/apps/cytohubba) (Tang et al., 2015) was used to calculate the topological properties of nodes in the network, and hub genes of GP were screened out in the treatment of T2DM. Subsequently, hub genes were used for functional and pathway enrichment analyses by means of the Database for A Gene Annotation & Analysis Resource (Metascape, metascape.org/gp/index.html). The compound-pathway-gene network was built by connecting the candidate compounds, the signaling pathways involved, and candidate targets.

### Western Blot Analyses

Pancreatic tissue was lysed with PMSF buffer (Soiarbio, Beijing, China) by means of an ultrasonic lysing instrument (30% w, 10 s, 5 s, six times; Lichen, Beijing, China). Lysates were denatured by heating for 10 min at 100 °C and loaded onto 12.5% SDS-polyacrylamide gels. Electrophoresis was performed at a constant voltage (80 V) for 90 min. Then proteins were transferred to polyvinylidene fluoride (PVDF) membranes (Millipore, Schwalbach, Germany). Membranes were blocked and probed with primary antibodies directed against iNS, AKT, eNOS, caspase-3, caspase-8, and MAPK in 1:1,000, followed by incubation with secondary horseradish peroxidase (HPR)–conjugated antibodies (1:2,000). Finally, membranes were incubated with an ECL kit for visualization (Yeason, Shanghai, China). The density of each band was quantified using Image Lab software (Qian et al., 2020).

## Results

### Metabolic Responses of Mice to GP Treatment

**FIGURE 1:**
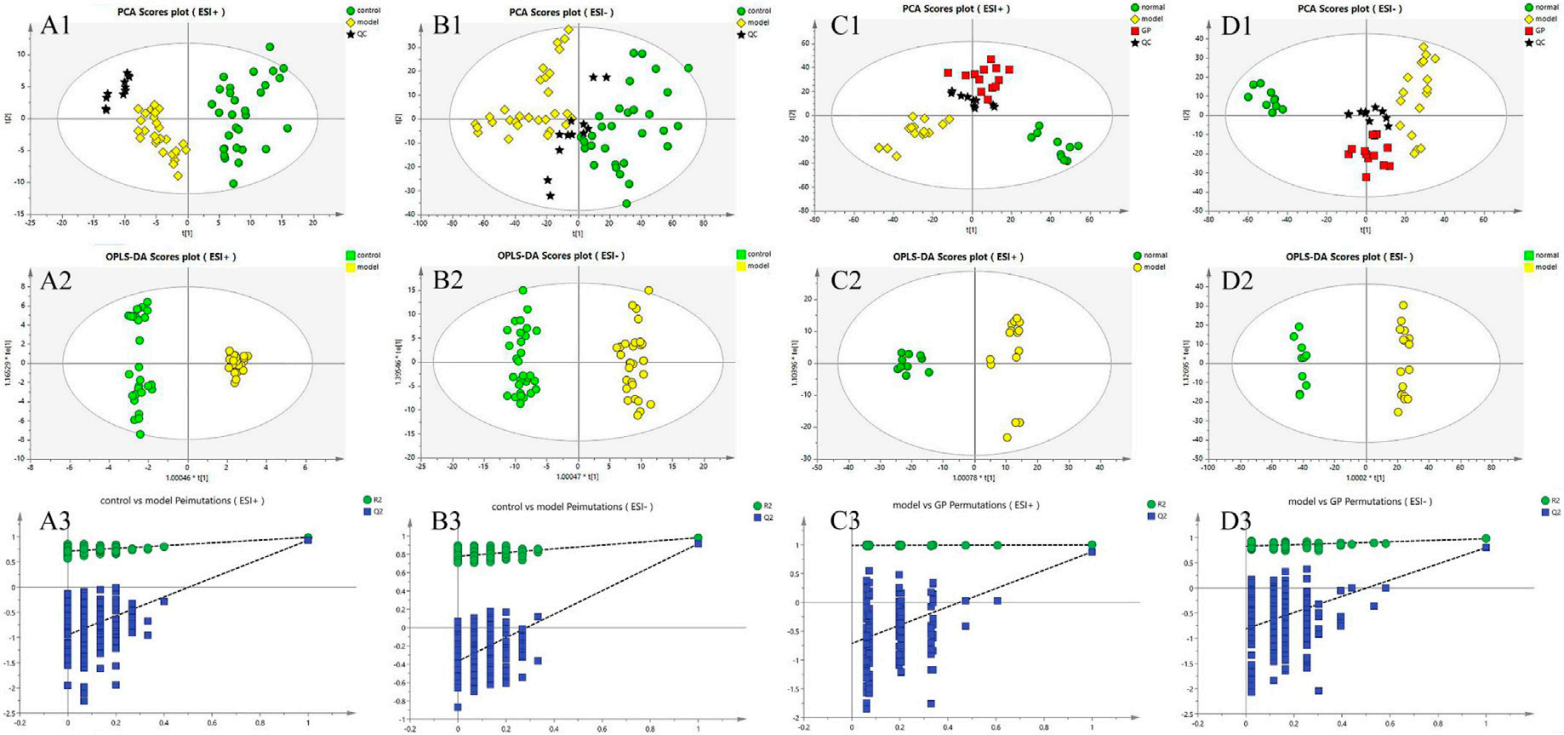
PCA and OPLS-DA and permutation score plots **(A1–A3)**: clinical plasma sample of the control and model group, ESI+; **(B1–B3)**: clinical sample of model and control, ESI; **(C1–C3)**: plasma samples in mice of the control, model, and GP groups, ESI+; **(D1–D3)**: plasma samples in mice of the control, model, and GP groups, ESI−.

In positive and negative ion modes, the peak intensity and retention time of typical ion peaks of QC samples are highly overlapping (Figure 1). The data quality met the requirements for statistical analysis, and the reproducibility of the methods was confirmed (Theodoridis et al., 2012).

PCA is an unsupervised method to observe metabolic differences among groups. The key metabolites of different regulations were identified by PCA analysis of LC-MS data of plasma samples. The PCA score map (Figures 1-A1 and B1) of clinical plasma samples in positive and negative ion modes was divided into two groups to show that there was a significant difference between the control group and the diabetic group. In the negative and positive ion modes of mouse plasma samples, the PCA score map (Figures 1-C1 and D1) showed that the control group and the model group were obviously divided into two groups, which proved that there was a significant difference between the two groups. In addition, the GP treatment group fell between the normal group and the model group, which indicated that considering the overlap between groups. To identify different metabolites in each group and maximize the separation between groups, the supervised pattern recognition method OPLS-DA was applied. OPLS-DA analysis in positive and negative ion modes (Figures 1-A2 and B2) showed that the two groups of clinical plasma samples were separated from each other and that each group could be well gathered together; OPLS-DA analysis in negative ion and positive ion modes (Figures 1-C2 and D2) showed that the three groups were separated from each other and that each group could be well gathered together.

In ESI+ and ESI− modes and the signal responses of plasma metabolites were combined to explain their distribution. The results between every two modes indicated that there was a significant separation in the metabolites. There was no overfitting and there was good clustering in the samples of each group in both the PCA plots and OPLS-DA plots. R2Y and Q2Y were used to evaluate the quality of OPLS-DA. In this research, the R2Y and Q2Y values were 0.713 and 0.946 in positive ESI mode (Figure 1-A3) of clinical plasma samples and 0.778 and 0.405 in negative ESI mode (Figure 1-B3) of clinical plasma samples. In mouse plasma samples, the R2Y and Q2Y were 0.988 and 0.713 in positive ESI mode (Figure 1-C3) and 0.828 and 0.811 in negative ESI mode (Figure 1-D3). Thus, these results suggested that the quality of the OPLS-DA model had a high reliability.

### Identification of Potential Biomarkers

In the OPLS-DA model, a VIP score > 1 and *p* values < 0.05 were selected, obtained the precursor ions and MS/MS, fragments using UPLC-QTOF-MS/MS with a high resolution, then using the online database HMDB matched with information of metabolites. Qualified metabolites with an error between extracted quality value and experimental quality value of less than 10 ppm were identified as candidate biomarkers. Finally, 67 metabolites from clinical plasma samples (Supplementary Table S1) and 50 metabolites from *db*/*db* mouse plasma samples (Supplementary Table S2) were identified as candidate biomarkers.

### Metabolic Pathway Enrichment Analysis

**FIGURE 2:**
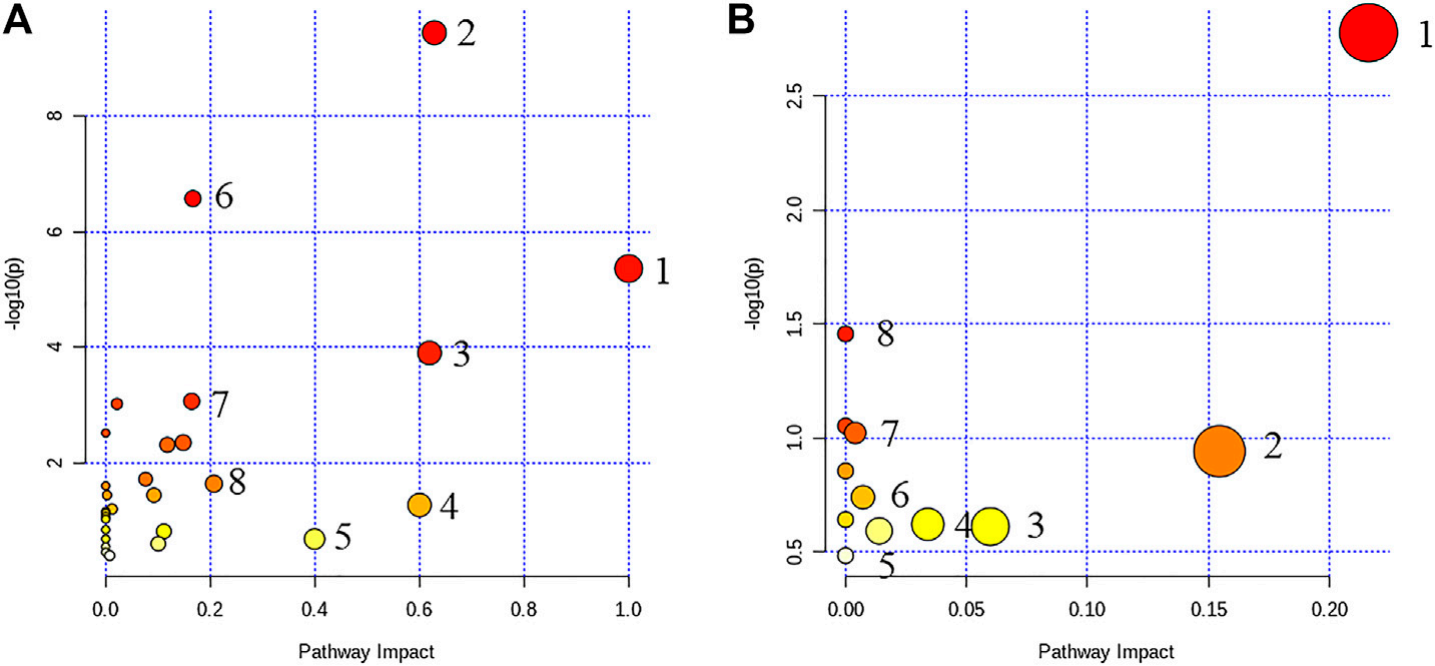
Overview of metabolic pathway analysis (**A**, clinical sample): 1) phenylalanine metabolism, 2) glycine, serine, and threonine metabolism, 3) retinol metabolism, 4) synthesis and degradation of ketone bodies, 5) beta-alanine metabolism, 6) aminoacyl-tRNA biosynthesis, 7) tyrosine metabolism, and 8) pyruvate metabolism; (**B**, plasma samples in mice): 1) glycine, serine, and threonine metabolism, 2) retinol metabolism, 3) pyrimidine metabolism, 4) arginine and proline metabolism, 5) tryptophan metabolism, 6) glutathione metabolism, 7) glycosyl phosphatidyl inositol (GPI)–anchor biosynthesis, and 8) sphingolipid metabolism.

Through the enrichment of 67 and 50 metabolites in the two categories (Figure 2), a metabolism pathway with a high score was constructed. In clinical samples, the major metabolism pathways included in these metabolites were concluded, namely, phenylalanine metabolism, glycine, serine, and threonine metabolism, retinol metabolism, synthesis and degradation of ketone bodies, beta-alanine metabolism, aminoacyl-tRNA biosynthesis, tyrosine metabolism, and pyruvate metabolism. In *db*/*db* mouse samples, the major metabolism pathways included in these metabolites were concluded, namely glycine, serine, and threonine metabolism, retinol metabolism, pyrimidine metabolism, arginine, and proline metabolism, tryptophan metabolism, glutathione metabolism, glycosyl phosphatidyl inositol (GPI)-anchor biosynthesis, and sphingolipid metabolism. The results indicated that T2DM was most affected by the metabolic pathways glycerol phosphate metabolism and retinol metabolism. Thus, GP may be effective in the treatment of T2DM by affecting these two metabolic pathways.

### Network Pharmacology of GP in T2DM

#### Identification of Potential Targets

**FIGURE 3:**
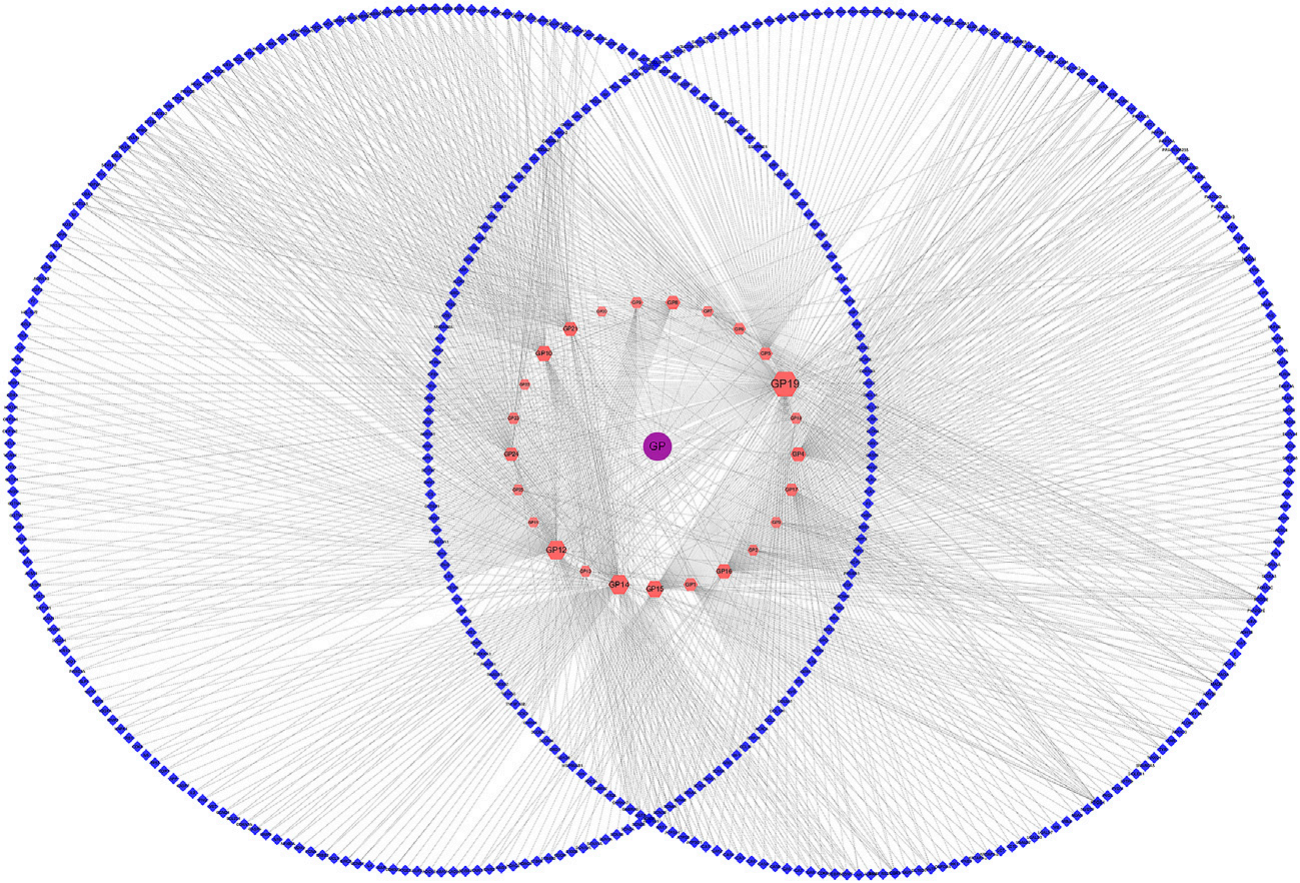
Compound–compound target network (blue nodes: predicted targets, red nodes: compound of *Gynura procumbens*, and purple nodes: drug of *Gynura procumbens* (GP)).

The STITCH, TCMSP, SwissTargetPrediction database, and DrugBank were applied to screen out 25 types of bioactive compounds (Supplementary Table S3) and 450 targets corresponding to the bioactive compounds of GP. The obtained bioactive compounds and targets were used for constructing a compound–compound target network (Figure 3), which consisted of 476 targets (25 bioactive compounds and 471 predicted targets) and 1,149 interaction edges. Among these compounds, quercetin (degree 213), luteolin (degree 125), kaempferol (degree 125), and hyperoside (degree 73) had the largest number of potential targets. Furthermore, GeneCards, TTD, ETCM, and OMIM databases were used to screen out 1,265 targets associated with T2DM (Zhu and Hou, 2020).

#### Protein–Protein Interaction Network for GP in the Treatment of T2DM and Hub Genes Analysis

**FIGURE 4:**
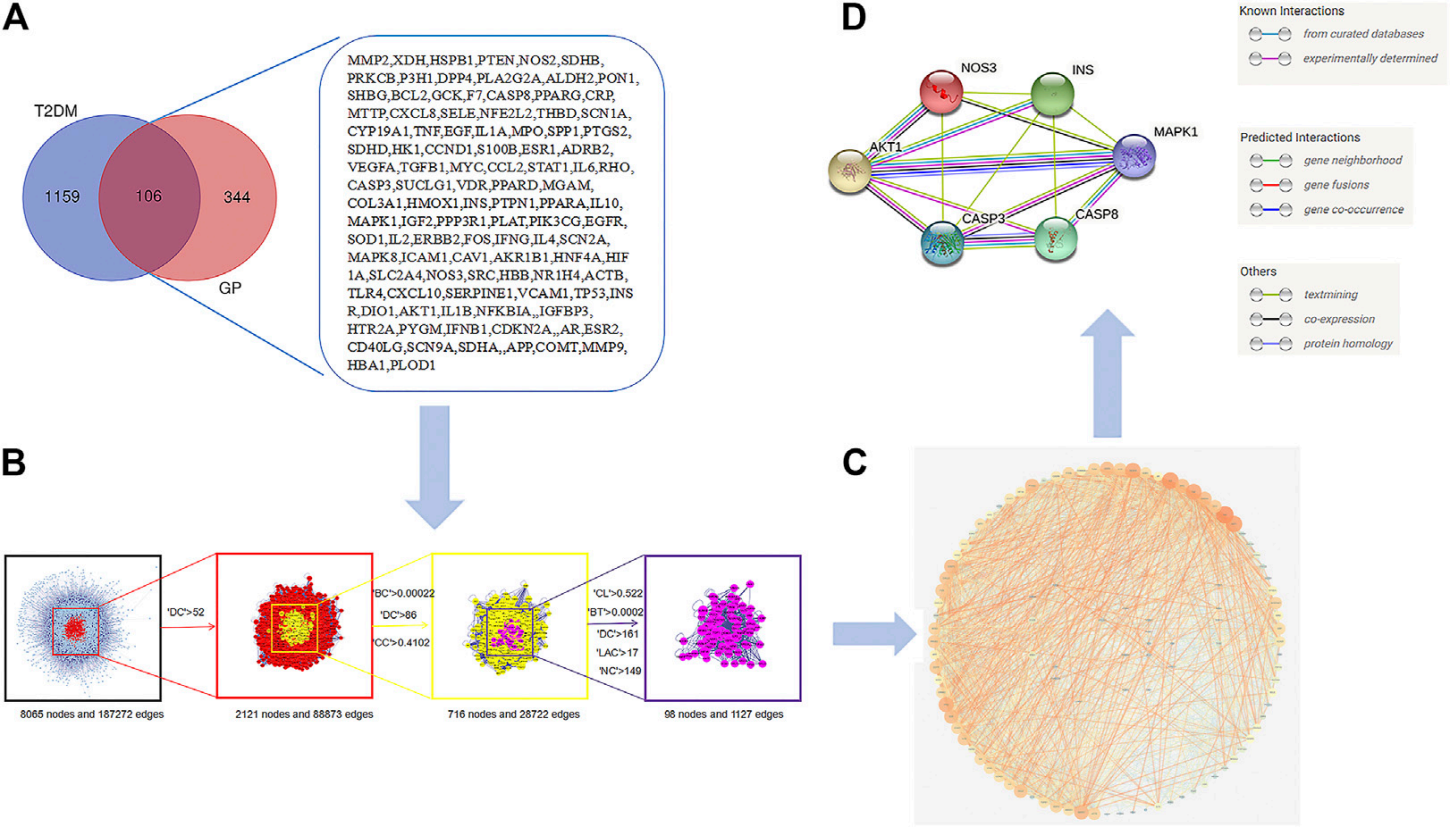
Hub genes of *Gynura procumbens* (GP) and type II diabetes mellitus (T2DM). **(A)** Venn diagram and candidate targets of *Gynura procumbens* (GP) and type II diabetes mellitus (T2DM), **(B)** arget filtering strategy diagram of hub nodes (degree centrality (DC), betweenness centrality (BC), closeness centrality (CC), network centrality (NC), local average connectivity (LAC)), **(C)** protein–protein interaction (PPI) network, and **(D)** Network of the top six hub genes.

Using a Venn diagram, 106 overlapped targets were screened out as the candidate targets of GP and T2DM (Figure 4A). The obtained targets were introduced into the STRING online database (protein–protein interaction (PPI) combined score > 0.7) to construct the PPI network presented in Figure 4B, consisting of 106 nodes and 1,796 interaction edges. Through BisoGenet and CytoHubba analysis, based on the degree centrality (DC), betweenness centrality (BC), closeness centrality (CC), network centrality (NC), local average connectivity (LAC), and screening of other indicators, a total of 98 “hub” nodes were identified. A schematic diagram of the screening strategy is shown in Figure 4C. To study the possible underlying mechanism of GP in the treatment of T2DM, ClueGO was used to analyze the abovementioned 98 hub nodes. The results showed regulation of cell cycle G2/M phase transition, regulation of mRNA processing, negative regulation of mRNA metabolic process, and predicted targets (Supplementary Figure S1). Based on the PPI network and hub ClueGO analysis, six hub targets were screened out: insulin (iNS), AKT serine/threonine kinase 1 (AKT1), mitogen-activated protein kinase 1 (MAPK1), nitric oxide synthase (NOS), endothelial (eNOS), caspase-3 (CASP3), and caspase-8 (CASP8) (Figure 4D; Supplementary Table S4).

#### Revealing Compound-Pathway-Gene Network and Analyses

**FIGURE 5:**
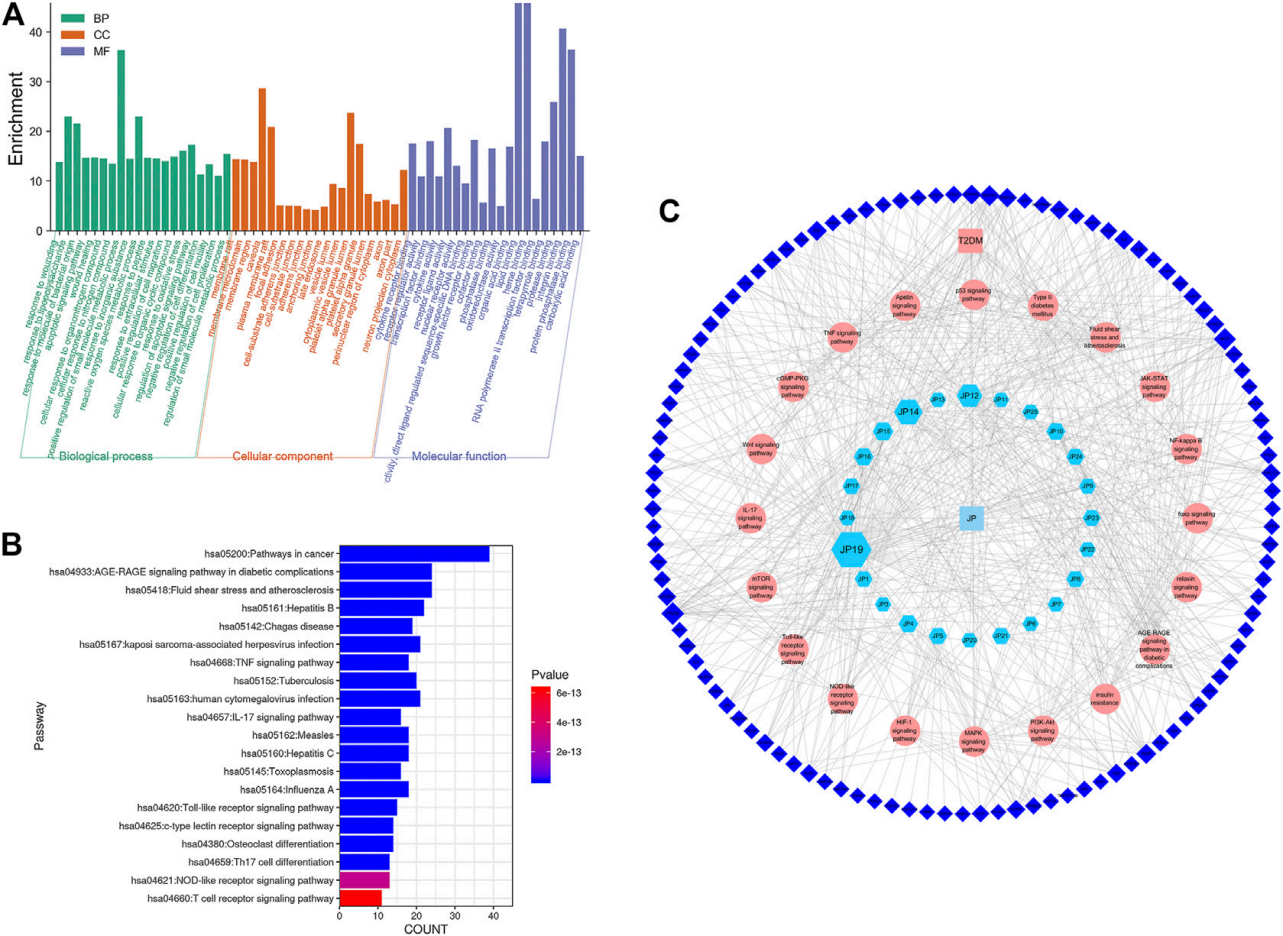
Main pathways of GP for T2DM. **(A)** GO enrichment analysis of targets of GP (*Gynura procumbens*) decoction, **(B)** Analysis of KEGG enrichment in 20 pathways as targets of GP (*Gynura procumbens*) decoction, and **(C)** component-pathway-gene network of GP for T2DM.

According to Figures 5A,B, GO and KEGG enrichment analyses of targets of the PPI network were performed using bioinformatics, and a compound-pathway-gene network was constructed (Figure 5C) to identify the potential pathway of T2DM that different GP compounds act upon. The network predicted that quercetin, luteolin, kaempferol, and phaseoloidin might be potential ingredients in GP and that PTGS2, AKT, MAPK1, TNF, INSR, IL6, MAPK8, PRKCB, NOS3, iNS, PIK3CG, BCL2, VEGFA, EGFR, CCND1, CASP3, and FOS might be potential targets of T2DM. The core targets were enriched in the regulation of mRNA processing, RNA polymerase, spindle assembly, hematopoietic stem cell differentiation, etc. The possible biological mechanisms involve the AGE–RAGE signaling pathway in diabetic complications, fluid shear stress, atherosclerosis, the MAPK signaling pathway, the PI3K–AKT signaling pathway, and HIF-1 signaling pathway; however, *in vitro* and *in vivo* studies are needed to verify these results.

#### Regulation of PI3K/AKT and AGE–RAGE Signaling *in vivo*

**FIGURE 6:**
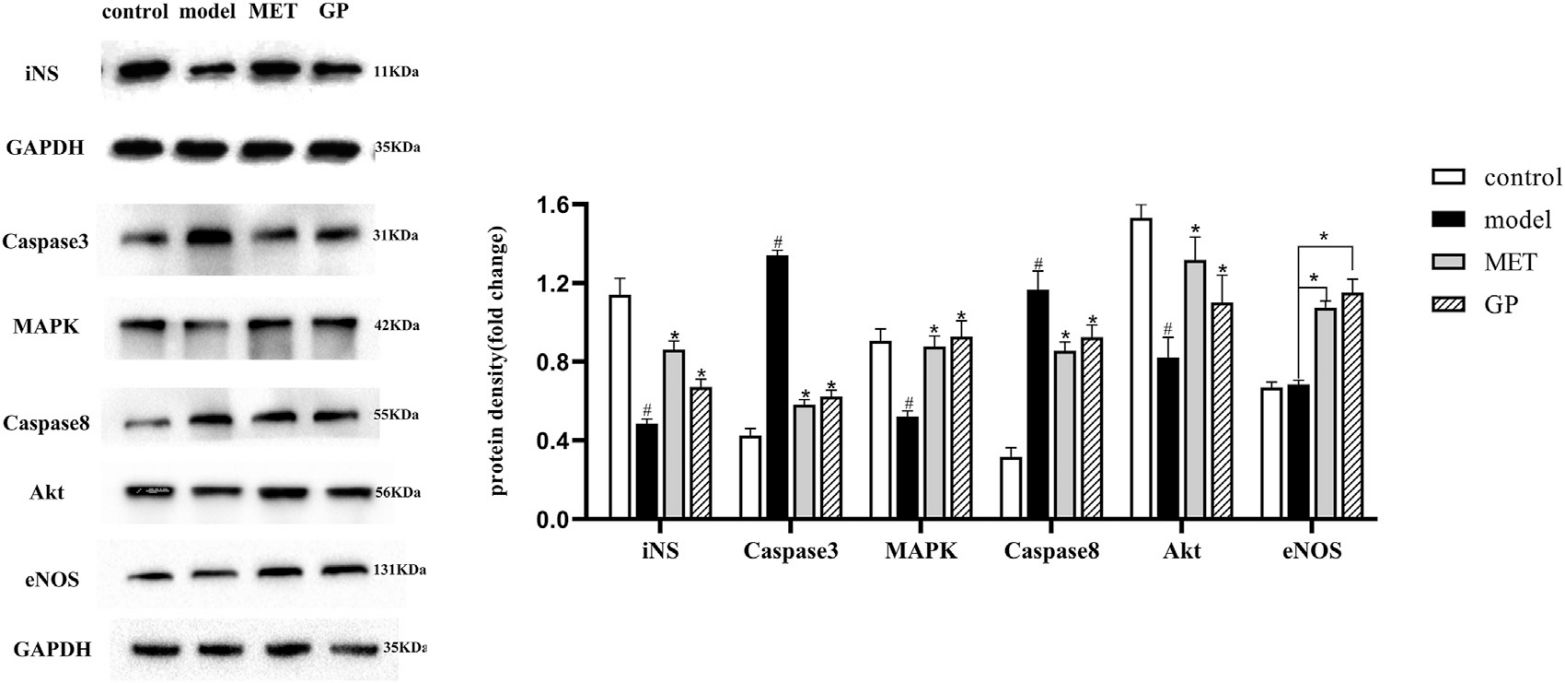
Effect of GP on protein expression in pancreatic tissue (iNS; caspase-3; MAPK; caspase-8; Akt; and eNOS) (control; model; MET; and GP). Data are expressed as the mean ± SD. “#” represents control group vs. model group (#*p* < 0.05, ##*p* < 0.01); “*” represents GP group, MET group vs. the model group (*p < 0.05, **p < 0.01).

To assess the underlying mechanism of GP in T2DM, PI3K/AKT and AGE–RAGE signaling pathways were investigated. The PI3K/AKT signaling pathway plays a crucial role in the regulation of glucose metabolism (Wu et al., 2021). During insulin resistance, to ensure that the blood sugar level stays within the normal level, islet B cells in pancreatic islets will secrete more insulin to make up for the lack of the hypoglycemic ability of unit insulin so as to ensure a normal blood sugar level. With the passage of time, the function of B cells is impaired and the compensatory ability of insulin secretion decreases, thereby resulting in impaired glucose tolerance and even T2DM. Hyperinsulinemia is one of the main features of metabolic syndrome, which is closely related to various complications of T2DM. In this study (Figure 6), AKT, iNS, eNOS, and MAPK were downregulated in the model group compared with the control group and significantly upregulated in the GP and MET groups compared with the model group. These results were in line with the data presented in previous reports (Alghanem et al., 2021; Cheng et al., 2021).

As reported, the AGE–RAGE signaling pathway is closely associated with T2DM and inflammation (Syed et al., 2020). In previous studies (Pan et al., 2015), it have been outlined that AGEs could lead to insulin resistance in adipocytes through impede insulin-mediated glucose transport and uptake (Walke et al., 2021a). These results showed that glycated insulin plays a part in insulin resistance by inflammatory pathways and impairing insulin signaling through the AGE–RAGE signaling pathway (Walke et al., 2021b). In this study, MAPK and eNOS were upregulated, while caspase-3 and caspase-8 were downregulated in the GP and MET groups compared with the model group. These results were consistent with the data presented in previous reports (Steven et al., 2017). Taken together, these results revealed that GP relies on PI3K/AKT and AGE–RAGE signaling to treat T2DM.

## Discussion

To our knowledge, this is the first study using network pharmacology technology and applying pathway enrichment approaches to dissect the molecular mechanisms of GP on T2DM from a network modulation point of view (Xue et al., 2013). The results suggested that the mechanisms of GP-treating T2DM might be related to the regulation of several metabolic- and disease-related signaling pathways. Based on these results, we concluded that the results of plasma metabonomics are likely to correspond with the network pharmacology technology results.

Moreover, based on the characteristics of multi-components and multi-targets of traditional Chinese medicine, in this study, using metabolomics technology, to enable high-throughput detection of metabolites and analysis of large datasets to identify, in thousands of metabolites, the phenotypic contribution is the greatest (Feng et al., 2019). The data showed that the common metabolic pathways of the clinical serum samples and the mouse plasma samples were glycine, serine, and threonine and retinol metabolisms. These results were consistent with classical metabolic pathways of T2DM reported to date (López-Hernández et al., 2019; Olsen and Blomhoff, 2020). It has been reported that through increasing lipid synthesis, oxidation, and sugar transport through insulin and the affinity for insulin receptors, polyunsaturated phospholipids could be associated with insulin resistance. On the other hand, serum retinol binding protein 4 (RBP4) must be combined with retinol to secrete effectively from hepatocytes, and retinol deficiency reduces serum levels of RBP4. The decrease in RBP4 secretion represents the decline in pancreatic function and aggravates insulin resistance (Chang et al., 2015). To sum up, the two metabolic pathways are all through reducing insulin resistance, protecting islet B cells, and inhibiting inflammation to treat T2DM. Based on these results, we concluded that the results of plasma metabonomics corresponded with the network pharmacology technology results. Therefore, we selected core targets (AKT, iNS, eNOS, MAPK, caspase-8, and caspase-3) in the PI3K/AKT and AGE–RAGE signaling pathways to verify the expression by Western blot analysis.

**FIGURE 7:**
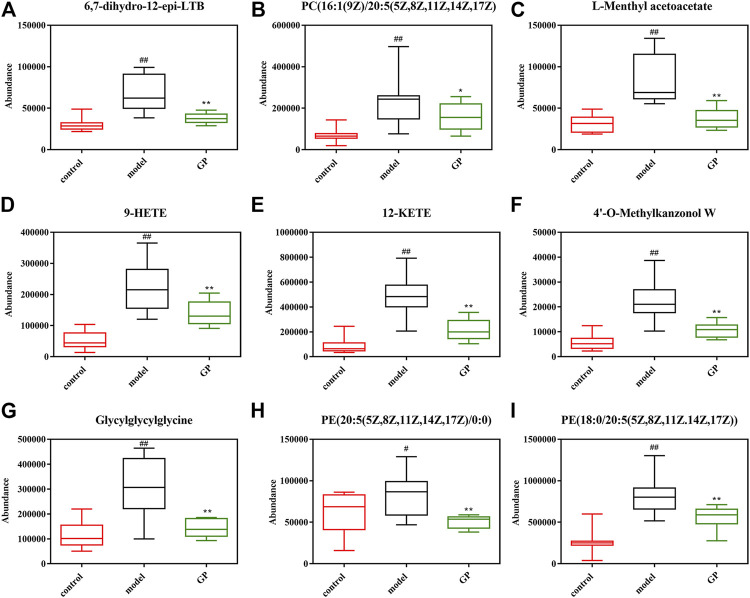
Relative levels of the selected 37 metabolites in the plasma of mice from the control, model, and GP groups. Data are expressed as the mean ± SD. “#” represents control group vs. model group (#*p* < 0.05, ##*p* < 0.01); “*” represents GP group vs. the model group (*p < 0.05, **p < 0.01).

Metabonomics analysis of mouse plasma after GP administration showed that GP had a significant callback effect on 37 metabolic components, of which the callback effect on nine biomarkers was obviously downregulated (6,7-dihydro-12-epi-LTB4, C (16:1(9Z)/20:5(5Z,8Z,11Z,14Z,17Z)), L-methyl acetoacetate, 9-HETE, 12-KETE, 4′-O-methylkanzonol W, glycylglycylglycine, LysoPE (20:5(5Z,8Z,11Z,14Z,17Z)/0:0), and PE (18:0/20:5(5Z,8Z,11Z,14Z,17Z)), which was very close to that of the normal control group (Figure 7). After further analysis of the callback metabolites, it was found that both the glycerol phosphate metabolism and retinol metabolism were most likely to be involved in the metabolism of glycerol phosphate and retinol. Metabonomics analysis of the two groups of plasma samples showed that the common metabolic pathways were glycerol phosphate metabolism and retinol metabolism (Xiaoming and Chunrong, 2017)^.^ Through studying targeted serum liposomes, it was found that several factors involved in glycerol phosphate metabolism were associated with a variety of cardiovascular disease risk factors. PC16; 0/2:0 and visceral fat negatively correlated with visceral fat, blood pressure, and fasting triacylglycerol, while PC14; 1/0:0 positively correlated with visceral fat, fasting insulin, and triglyceride. When investigating the serum metabolism group, several biomarkers involved in glycerol phosphate metabolism were found, and GP regulated the pathway by reducing the amount of the biomarkers in mouse serum so as to improve the disease and reduce the blood sugar level. In addition to the above metabolic pathways, some disease-related signaling pathways, glycosyl phosphatidyl inositol (GPI)–anchor biosynthesis, pyrimidine metabolism, arginine and proline metabolism, and regulation of tryptophan channels by inflammatory mediators were found to be regulated by GP extracts. For other metabolic pathways related to T2DM found in this study, we planned to study further in the future.

Traditional herbal medicine has many components and can act on many targets. Because of the complexity of herbs, it is one-sided to use only one method to determine the active substance and potential mechanism of action. Therefore, it is necessary to develop a comprehensive strategy to help people deeply understand the overall and collaborative nature of herbal medicine. In order to reveal the antidiabetic effect, potential mechanism, and active components of GP, we combined plasma metabolomics, network analysis, and Western blot analysis successfully. The obtained results suggested that GP has significant antidiabetic effects. The potential targets and active components concluded, providing indispensable information for the quality control and drug development of GP. In addition, the network pharmacology technology concluded that quercetin and luteolin are potential active substances in GP for the treatment of T2DM, which provides a basis for novel drugs to treat T2DM. Our findings provide a novel methodological reference to reveal the active ingredients and regulatory mechanisms of herbal medicines.

## Conclusion

GP has great potential for treating T2DM by inhibiting insulin resistance, promoting insulin production, protecting islet B cells, and improving inflammation. In addition, the metabolic disorder caused by diabetes can be partially reversed by GP treatment. Integration pathway analysis revealed two metabolic pathways (glycerol phosphate metabolism and retinol metabolism) and disease-related protein signaling pathways (PI3K/AKT and AGE–RAGE) were significantly associated with the antidiabetic effects of GP. In addition, selecting MET as the positive control drug, Western blot analysis results indicated that the protein expression of AKT, eNOS, iNS, and MAPK was activated and significantly upregulated, while caspase-8 and caspase-3 were significantly downregulated in the GP and MET groups compared with the model group. Our findings indicated that GP, by regulating glycerol phosphate metabolism and retinol metabolism to reduce insulin resistance, protected islet B cells and inhibited inflammation to treat T2DM. In conclusion, our findings provide a theoretical basis for further development and application of GP.

## Data Availability

The raw data supporting the conclusions of this article will be made available by the authors, without undue reservation, to any qualified researcher.
