# Supplementary figures and images for: Based on Plasma Metabonomics and Network Pharmacology Exploring the Therapeutic Mechanism of Gynura procumbens on Type 2 Diabetes
Source: Front Pharmacol. 2021 May 28;12:674379. doi: 10.3389/fphar.2021.674379 (PMC8192979; doi:10.3389/fphar.2021.674379)

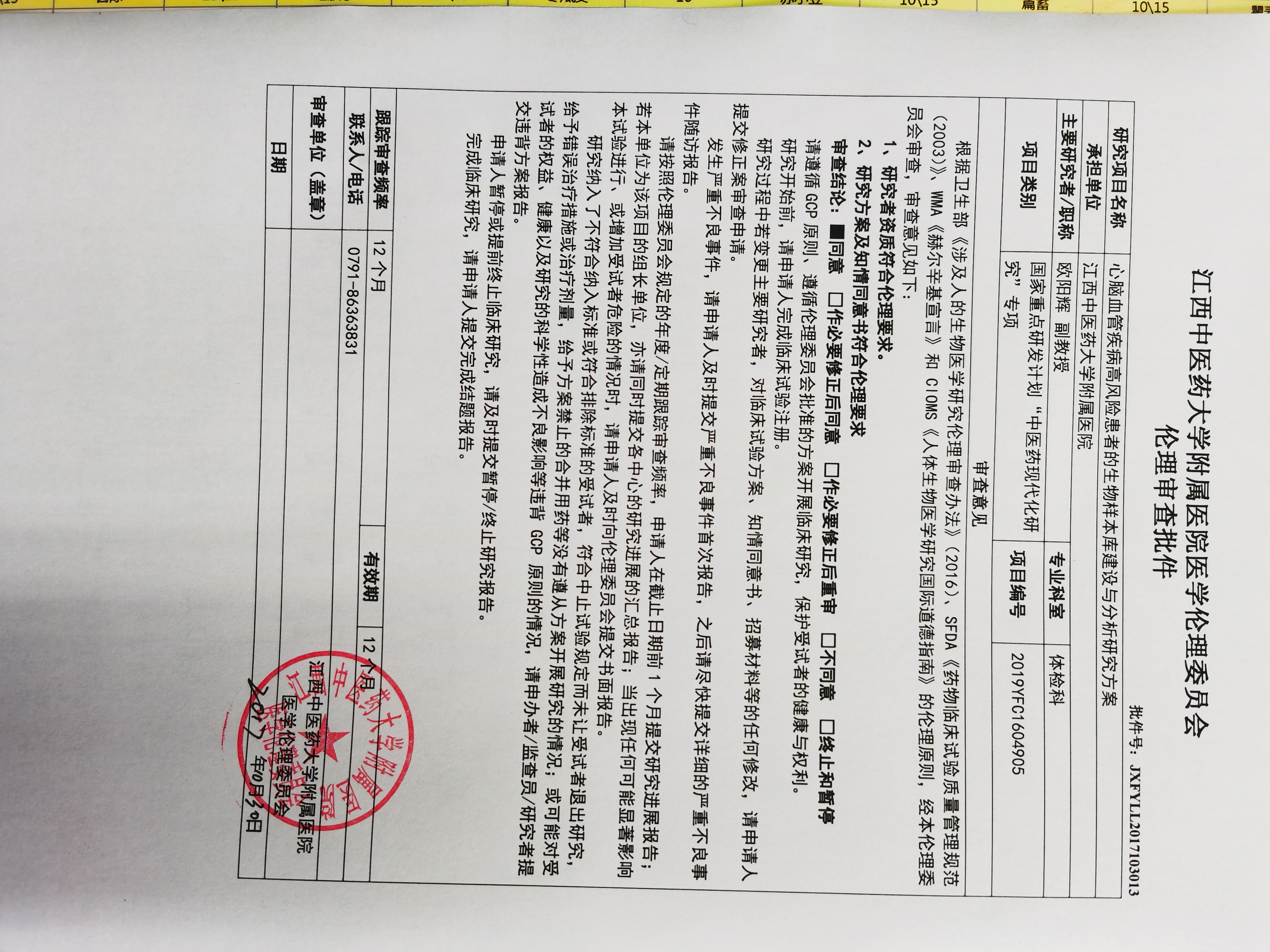

Supplement: Supplementary file 1 [file Image1.JPEG]
