# Supplementary material for: Based on Plasma Metabonomics and Network Pharmacology Exploring the Therapeutic Mechanism of Gynura procumbens on Type 2 Diabetes
Source: Front Pharmacol. 2021 May 28;12:674379. doi: 10.3389/fphar.2021.674379 (PMC8192979; doi:10.3389/fphar.2021.674379)
Supplement: Supplementary file 2 [file DataSheet1.docx]

**List of Legends**

**Table S1.** Potential biomarkers of clinicial plasma in control and model group

**Table S2.** Potential biomarkers of type 2 diabetic post-treatment with GEB

**Table S3.** Basic information of bioactive compounds of Gynura procumbens

**Table S4.** Hub genes of treating type 2 diabetic of Gynura procumbens Prescription and its topological properties

**Table S1.** Potential biomarkers of clinicial plasma in control and model group

| NO | RT | Name | M.F. | Extraction Mass(Da) | Experimental mass | Ion Mode | Erro (ppm) | HMDB | adjust |
| --- | --- | --- | --- | --- | --- | --- | --- | --- | --- |
| P1 | 8.81 | Arachidonic acid | C20H32O2 | 305.24751 | 304.24023 | [M+H]+ | 2.4 | HMDB0001043 | ↓ |
| P2 | 22.02 | Docosahexaenoic acid | C22H32O2 | 329.24751 | 328.24023 | [M+H]+ | 0.6 | HMDB0002183 | ↓ |
| P4 | 8.2 | Docosapentaenoic acid (22n-6) | C22H34O2 | 331.26316 | 330.25588 | [M+H]+ | 4.1 | HMDB0001976 | ↓ |
| P5 | 7.66 | Palmitoleic acid | C16H30O2 | 255.23186 | 254.22458 | [M+H]+ | 2.5 | HMDB0003229 | ↓ |
| P6 | 7.62 | Eicosapentaenoic acid | C20H30O2 | 303.23186 | 302.22458 | [M+H]+ | 2.2 | HMDB0001999 | ↓ |
| P7 | 10.74 | Sulfolithocholylglycine | C26H43NO7S | 514.2833 | 513.27603 | [M+H]+ | 2.8 | HMDB0002639 | ↓ |
| P8 | 8.22 | Adrenic acid | C22H36O2 | 333.27881 | 332.27153 | [M+H]+ | 2.1 | HMDB0002226 | ↓ |
| P9 | 11.7 | Arachidonoylglycerophosph oinositol | C29H49O12P | 621.30344 | 620.29617 | [M+H]+ | 5.8 | HMDB0000679 | ↓ |
| P10 | 1.34 | Phenylpyruvic acid | C9H8O3 | 165.05462 | 164.04734 | [M+H]+ | 3.9 | HMDB0000205 | ↓ |
| P11 | 2.96 | 3-Indolepropionic acid | C11H11NO2 | 190.08626 | 189.07898 | [M+H]+ | -4.6 | HMDB0000396 | ↓ |
| P12 | 10.04 | 9,10-Epoxyoctadecenoic acid | C18H32O3 | 297.24242 | 296.23515 | [M+H]+ | 4.3 | HMDB0004701 | ↑ |
| P13 | 11.81 | Kinetensin 4-7 | C26H37N9O6 | 572.29396 | 571.28668 | [M+H]+ | 9.1 | HMDB0012986 | ↑ |
| P15 | 4.19 | 12-Ketodeoxycholic acid | C24H38O4 | 391.28429 | 390.27701 | [M+H]+ | 4.5 | HMDB0000328 | ↑ |
| P16 | 4.48 | 7 alpha-Hydroxy-3-oxo-4-cholestenoate | C27H42O4 | 431.31559 | 430.30831 | [M+H]+ | 7 | HMDB0012458 | ↑ |
| P17 | 8.9 | 1- glycero-3-phosphate | C23H41O7P | 461.26627 | 460.25899 | [M+H]+ | -2.8 | HMDB0114746 | ↑ |
| P18 | 8.81 | Dihomo-gamma-linolenic acid | C20H34O2 | 307.26316 | 306.25588 | [M+H]+ | 4.9 | HMDB0002925 | ↑ |
| P19 | 9.82 | 1-docosahexaenoyl-glycero-3-phosphate | C25H39O7P | 483.25062 | 482.24334 | [M+H]+ | 0.6 | HMDB0114755 | ↑ |
| P20 | 10.32 | LysoPC (20:3) | C28H52NO7P | 546.35542 | 545.34814 | [M+H]+ | 9.3 | HMDB0010393 | ↑ |
| P21 | 4.72 | LysoPE (0:0/18:2) | C23H44NO7P | 478.29282 | 477.28554 | [M+H]+ | 2.7 | HMDB0011477 | ↑ |
| P22 | 22.05 | Oleic acid | C18H34O2 | 283.26316 | 282.25588 | [M+H]+ | 2.6 | HMDB0000207 | ↑ |
| P23 | 9.89 | LysoPC (18:2) | C26H50NO7P | 520.33977 | 519.33249 | [M+H]+ | 5 | HMDB0010386 | ↑ |
| P24 | 7.62 | Retinyl ester | C20H30O2 | 303.23186 | 302.22458 | [M+H]+ | 2.2 | HMDB0003598 | ↓ |
| P25 | 22.08 | Spermidine | C7H19N3 | 146.16517 | 145.1579 | [M+H]+ | -6.5 | HMDB0001257 | ↓ |
| P26 | 10.43 | PC(20:4(5Z,8Z,11Z,14Z)/16:1(9Z)) | C44H78NO8P | 780.55378 | 779.54651 | [M+H]+ | 3.8 | HMDB0008430 | ↓ |
| P27 | 10.43 | PC(18:2(9Z,12Z)/22:6(4Z,7Z,10Z,13Z,16Z,19Z)) | C48H82NO8P | 832.58508 | 831.57781 | [M+H]+ | -0.9 | HMDB0008156 | ↓ |
| P28 | 10.43 | PC(20:1(11Z)/20:5(5Z,8Z,11Z,14Z,17Z)) | C48H84NO8P | 834.60073 | 833.59346 | [M+H]+ | 9 | HMDB0008314 | - |
| P29 | 10.43 | PC(15:0/20:3(8Z,11Z,14Z)) | C43H80NO8P | 770.56943 | 769.56216 | [M+H]+ | 8.3 | HMDB0007948 | - |
| P30 | 10.43 | PC(16:0/20:3(5Z,8Z,11Z)) | C44H82NO8P | 784.58508 | 783.57781 | [M+H]+ |  | HMDB0007980 | ↓ |
| P31 | 10.43 | PC(18:3(6Z,9Z,12Z)/22:4(7Z,10Z,13Z,16Z)) | C48H82NO8P | 832.58508 | 831.57781 | [M+H]+ | -0.9 | HMDB0008186 | ↓ |
| P32 | 22.08 | LysoPC(20:4(5Z,8Z,11Z,14Z)) | C28H50NO7P | 544.33977 | 543.33249 | [M+H]+ | 3.6 | HMDB0010395 | - |
| P33 | 16.92 | LysoPC(20:0/0:0) | C28H58NO7P | 552.40237 | 551.39509 | [M+H]+ | 9.5 | HMDB0010390 | - |
| P34 | 10.43 | LysoPE(20:5(5Z,8Z,11Z,14Z,17Z)/0:0) | C25H42NO7P | 500.27717 | 499.26989 | [M+H]+ | 3.5 | HMDB0011519 | - |
| P35 | 10.32 | LysoPC(20:3(8Z,11Z,14Z)) | C28H52NO7P | 546.35542 | 545.34814 | [M+H]+ | 9.3 | HMDB0010394 | - |
| P36 | 7.7 | 4,6-Dimethylnonanoic acid | C12H21NO | 196.16959 | 195.16231 | [M+H]+ | 17 | HMDB0002373 | - |
| P37 | 7.7 | 4,5-Dimethyl-2-heptyloxazole | C12H21NO | 196.16959 | 195.16231 | [M+H]+ | 17 | HMDB0037902 | - |
| P38 | 7.83 | Artemisyl propionate | C13H22O2 | 211.16926 | 210.16198 | [M+H]+ | 0.8 | HMDB0041554 | - |
| P39 | 7.7 | 5-Ethyl-2-hexyl-4-methyloxazole | C12H21NO | 196.16959 | 195.16231 | [M+H]+ | 17 | HMDB0037899 | - |
| P40 | 7.7 | 4-Methyl-2-pentyl-5-propyloxazole | C12H21NO | 196.16959 | 195.16231 | [M+H]+ | 17 | HMDB0037900 | - |
| P41 | 7.83 | Tsibulin 1 | C13H22O2 | 211.16926 | 210.16198 | [M+H]+ | 0.8 | HMDB0037390 | - |
| P42 | 7.83 | Linalyl propionate | C13H22O2 | 211.16926 | 210.16198 | [M+H]+ | 0.8 | HMDB0030425 | - |
| P43 | 7.83 | 2-Propenyl cyclohexanebutanoate | C13H22O2 | 211.16926 | 210.16198 | [M+H]+ | 0.8 | HMDB0038291 | - |
| P44 | 7.83 | Methyl (2E,6Z)-dodecadienoate | C13H22O2 | 211.16926 | 210.16198 | [M+H]+ | 0.8 | HMDB0031014 | - |
| P45 | 7.83 | Isobornyl propionate | C13H22O2 | 211.16926 | 210.16198 | [M+H]+ | 0.8 | HMDB0038249 | - |
| P46 | 7.83 | alpha-Terpineol propanoate | C13H22O2 | 211.16926 | 210.16198 | [M+H]+ | 0.8 | HMDB0032052 | - |
| P47 | 7.83 | Citral propylene glycol acetal | C13H22O2 | 211.16926 | 210.16198 | [M+H]+ | 0.8 | HMDB0037286 | - |
| P48 | 22.04 | 4-Ethyl-2-hexyl-5-methyloxazole | C12H21NO | 196.16959 | 195.16231 | [M+H]+ | 17 | HMDB0037901 | - |
| P49 | 7.83 | Propyl 2,4-decadienoate | C13H22O2 | 211.16926 | 210.16198 | [M+H]+ | 0.8 | HMDB0037307 | - |
| P50 | 7.83 | Cepanone | C13H22O2 | 211.16926 | 210.16198 | [M+H]+ | 0.8 | HMDB0032098 | - |
| P51 | 22.09 | 7-Ethyl-3,6-dihydro-1,4-dimethylazulene | C14H18 | 187.14813 | 186.14085 | [M+H]+ | 6 | HMDB0036471 | - |
| P52 | 22.09 | 7-Ethyl-5,6-dihydro-1,4-dimethylazulene | C14H18 | 187.14813 | 186.14085 | [M+H]+ | 6 | HMDB0035759 | - |
| P53 | 22.04 | 2-hydroxymexiletine | C11H17NO2 | 196.13321 | 195.12593 | [M+H]+ | 13.1 | HMDB0060953 | - |
| P54 | 7.83 | Dihydro-3-(2-octenyl)-2,5-furandione | C12H18O3 | 211.13287 | 210.12559 | [M+H]+ | 3.9 | HMDB0033102 | - |
| P55 | 22.08 | Spermidine | C7H19N3 | 146.16517 | 145.1579 | [M+H]+ | -6.5 | HMDB0001257 | - |
| P56 | 22.08 | LysoPC(20:4(5Z,8Z,11Z,14Z)) | C28H50NO7P | 544.33977 | 543.33249 | [M+H]+ | 3.6 | HMDB0010395 | ↑ |
| P57 | 10.32 | LysoPC(20:3(8Z,11Z,14Z)) | C28H52NO7P | 546.35542 | 545.34814 | [M+H]+ | 9.3 | HMDB0010397 | - |
| P58 | 22.04 | L-Menthyl acetoacetate | C14H24O3 | 241.17982 | 240.17254 | [M+H]+ | 8.2 | HMDB0032369 | - |
| P59 | 10.44 | PE(18:0/20:5(5Z,8Z,11Z,14Z,17Z)) | C43H76NO8P | 766.53813 | 765.53086 | [M+H]+ | 9.2 | HMDB0009005 | - |
| P60 | 2.96 | Indole-3-propionic acid | C11H11NO2 | 190.08626 | 189.07898 | [M+H]+ | -4.6 | HMDB0002302 | ↓ |
| P61 | 5.53 | DG(42:5) | C45H78O5 | 699.5922 | 698.58493 | [M+H]+ | 0 | HMDB0056080 | ↓ |
| P62 | 10.43 | PS(O-18:0/18:2) | C42H80NO9 P | 774.56435 | 773.55707 | [M+H]+ | 4.3 | HMDB0012380 | ↑ |
| P63 | 10.43 | PC(18:0_22:6) | C48H84NO8P | 834.60073 | 833.59346 | [M+H]+ | 9 | HMDB0008123 | ↑ |
| P64 | 5.78 | MG(26:4) | C29H50O4 | 463.37819 | 462.37091 | [M+H]+ | -8 | HMDB0072856 | ↑ |
| P65 | 9.84 | LPC(20:3) | C28H52NO7P | 546.35542 | 545.34814 | [M+H]+ | 9.3 | HMDB0010393 | ↑ |
| P66 | 9.91 | LPC(20:3) | C28H50NO7P | 544.33977 | 543.33249 | [M+H]+ | 3.6 | HMDB0010394 | - |
| P67 | 9.91 | LPC(20:4) | C28H50NO7P | 544.33977 | 543.33249 | [M+H]+ | 3.6 | HMDB0010395 | - |

**Table S2.** Potential biomarkers of type 2 diabetic post-treatment with GEB

| NO | RT | NAME | M.F. | Ion Mode | Extraction mass | Experimental mass | Error (ppm) | HMDB | adjust |
| --- | --- | --- | --- | --- | --- | --- | --- | --- | --- |
| 1 | 10.09 | Retinyl ester | C20H30O2 | [M+H]+ | 303.2317 | 303.2319 | 0.1 | HMDB0003598 | ↓ |
| 2 | 13.06 | Spermidine | C7H19N3 | [M+H]+ | 145.2459 | 146.1645 | -4.4 | HMDB0001257 | ↓ |
| 3 | 1.74 | Indoleacetaldehyde | C10H9NO | [M+H]+ | 160.0758 | 160.075 | -4 | HMDB0001190 | ↑ |
| 4 | 11.76 | N-Oleoylethanolamine | C20H39NO2 | [M+H]+ | 326.3039 | 326.3053 | -0.2 | HMDB0002088 | ↓ |
| 5 | 12.36 | 6,7-dihydro-12-epi-LTB4 | C20H34O4 | [M+H]+ | 339.2545 | 339.2526 | -1.2 | HMDB0012838 | ↓ |
| 6 | 21.01 | PC(18:0/20:4(5Z,8Z,11Z,14Z)) | C46H84NO8P | [M+H]+ | 810.5961 | 810.6002 | -0.7 | HMDB0008048 | ↓ |
| 7 | 19.57 | PC(20:4(5Z,8Z,11Z,14Z)/16:1(9Z)) | C44H78NO8P | [M+H]+ | 780.546 | 780.55 | -4.9 | HMDB0008430 | ↓ |
| 8 | 18.53 | PC(20:5(5Z,8Z,11Z,14Z,17Z)/18:3(9Z,12Z,15Z)) | C46H76NO8P | [M+H]+ | 802.5303 | 802.5371 | -1.3 | HMDB0008502 | ↓ |
| 9 | 21.01 | PC(20:4(5Z,8Z,11Z,14Z)/18:1(9Z)) | C46H82NO8P | [M+H]+ | 808.5827 | 808.5834 | -2 | HMDB0008433 | ↓ |
| 10 | 15.8 | PC(20:4(5Z,8Z,11Z,14Z)/20:5(5Z,8Z,11Z,14Z,17Z)) | C48H78NO8P | [M+H]+ | 829.5616 | 828.5505 | -4 | HMDB0008445 | ↓ |
| 11 | 21.91 | PC(18:2(9Z,12Z)/22:6(4Z,7Z,10Z,13Z,16Z,19Z)) | C48H82NO8P | [M+H]+ | 830.5726 | 832.5838 | -1.6 | HMDB0008156 | ↓ |
| 12 | 20.76 | PC(20:1(11Z)/20:5(5Z,8Z,11Z,14Z,17Z)) | C48H84NO8P | [M+H]+ | 834.6031 | 834.6001 | -0.8 | HMDB0008314 | - |
| 13 | 17.71 | PC(18:3(6Z,9Z,12Z)/20:5(5Z,8Z,11Z,14Z,17Z)) | C46H76NO8P | [M+H]+ | 802.5369 | 802.5371 | -1.3 | HMDB0008182 | ↓ |
| 14 | 20.91 | PC(20:3(5Z,8Z,11Z)/18:3(9Z,12Z,15Z)) | C46H80NO8P | [M+H]+ | 806.564 | 806.5686 | -1 | HMDB0008371 | - |
| 15 | 21.29 | PC(14:0/18:1(11Z)) | C40H78NO8P | [M+H]+ | 732.5494 | 732.5534 | -0.5 | HMDB0007872 | ↓ |
| 16 | 20.73 | PC(15:0/18:2(9Z,12Z)) | C41H78NO8P | [M+H]+ | 744.5486 | 744.5538 | 0.1 | HMDB0007940 | - |
| 17 | 21.95 | PC(18:0/18:3(6Z,9Z,12Z)) | C44H82NO8P | [M+H]+ | 766.5704 | 784.5844 | -0.8 | HMDB0008040 | ↓ |
| 18 | 18.96 | PC(15:0/20:4(8Z,11Z,14Z,17Z)) | C43H78NO8P | [M+H]+ | 768.5527 | 768.5531 | -0.9 | HMDB0007950 | ↓ |
| 19 | 20.17 | PC(15:0/20:3(8Z,11Z,14Z)) | C43H80NO8P | [M+H]+ | 770.5678 | 770.5698 | 0.4 | HMDB0007948 | - |
| 20 | 16.07 | PC(16:1(9Z)/20:5(5Z,8Z,11Z,14Z,17Z)) | C44H76NO8P | [M+H]+ | 778.5374 | 778.5373 | -1.1 | HMDB0008017 | ↓ |
| 21 | 21.16 | PC(18:1(11Z)/18:3(9Z,12Z,15Z)) | C44H80NO8P | [M+H]+ | 782.5636 | 782.5687 | -1 | HMDB0008074 | - |
| 22 | 21 | PC(16:0/20:3(5Z,8Z,11Z)) | C44H82NO8P | [M+H]+ | 784.584 | 784.5844 | -0.8 | HMDB0007980 | ↓ |
| 23 | 10.13 | PC(18:0/18:2(9Z,12Z)) | C44H84NO8P | [M+H]+ | 786.5957 | 786.6001 | -0.9 | HMDB0008039 | ↓ |
| 24 | 16.32 | PC(20:4(8Z,11Z,14Z,17Z)/18:4(6Z,9Z,12Z,15Z)) | C46H76NO8P | [M+H]+ | 806.5664 | 802.5371 | -1.3 | HMDB0008470 | ↓ |
| 25 | 18.71 | PC(18:3(6Z,9Z,12Z)/22:4(7Z,10Z,13Z,16Z)) | C48H82NO8P | [M+H]+ | 832.5831 | 832.5838 | -1.6 | HMDB0008186 | ↓ |
| 26 | 10.68 | LysoPC(18:1(9Z)) | C26H52NO7P | [M+H]+ | 522.3526 | 522.3544 | -2 | HMDB0002815 | - |
| 27 | 9.81 | LysoPC(20:4(5Z,8Z,11Z,14Z)) | C28H50NO7P | [M+H]+ | 544.3354 | 544.3394 | -0.7 | HMDB0010395 | - |
| 28 | 9.74 | LysoPC(20:2(11Z,14Z)) | C28H54NO7P | [M+H]+ | 548.3685 | 548.3704 | -1.2 | HMDB0010392 | - |
| 29 | 12.61 | LysoPC(20:0/0:0) | C28H58NO7P | [M+H]+ | 552.4001 | 552.4015 | -1.6 | HMDB0010390 | - |
| 30 | 8.98 | LysoPE(20:5(5Z,8Z,11Z,14Z,17Z)/0:0) | C25H42NO7P | [M+H]+ | 500.2749 | 500.2767 | -0.9 | HMDB0011519 | - |
| 31 | 11.53 | LysoPC(20:3(8Z,11Z,14Z)) | C28H52NO7P | [M+H]+ | 546.3504 | 546.3545 | -1.7 | HMDB0010394 | - |
| 32 | 8.86 | L-Menthyl acetoacetate | C14H24O3 | [M-H]- | 239.1645 | 239.1653 | 0.2 | HMDB0032369 | ↓ |
| 33 | 10.43 | 3-Oxohexadecanoic acid | C16H30O3 | [M-H]- | 269.2119 | 269.2121 | -0.5 | HMDB0010733 | ↓ |
| 34 | 11.3 | 9-HETE | C20H32O3 | [M-H]- | 301.217 | 301.2165 | 0.8 | HMDB0010222 | ↓ |
| 35 | 12.92 | (13R,14R)-7-Labdene-13,14,15-triol | C20H36O3 | [M-H]- | 305.2483 | 305.2483 | 2.7 | HMDB0034958 | ↓ |
| 36 | 9.53 | 12-KETE | C20H30O3 | [M-H]- | 317.2116 | 317.2123 | 0.1 | HMDB0013633 | ↓ |
| 37 | 11.75 | Methyl-[10]-shogaol | C22H32O2 | [M-H]- | 327.2329 | 327.2328 | -0.6 | HMDB0031465 | ↓ |
| 38 | 10.81 | Ginkgoic acid | C22H34O3 | [M-H]- | 345.2441 | 345.2438 | 0.7 | HMDB0033897 | ↓ |
| 39 | 10.72 | 4'-O-Methylkanzonol W | C18H22O5S | [M-H]- | 349.1114 | 349.1104 | -3.3 | HMDB0032602 | ↓ |
| 40 | 11.75 | Calcitroic acid | C24H30N4 | [M-H]- | 373.2403 | 373.2385 | -3.4 | HMDB0006472 | ↓ |
| 41 | 11.43 | Glycylglycylglycine | C6H11N3O4 | [M-H]- | 377.143 | 188.0722 | -0.7 | HMDB0029419 | ↓ |
| 42 | 11.93 | 5-Hexyltetrahydro-2-furanoctanoic acid | C18H34O3 | [M-H]- | 297.2442 | 297.2434 | -0.5 | HMDB0031127 | ↓ |
| 43 | 11.43 | 1-Formylneogrifolin | C23H32O3 | [M-H]- | 355.2251 | 355.2279 | 0 | HMDB0039880 | ↓ |
| 44 | 6.17 | 3-Hydroxyvalproic acid | C8H16O3 | [M-H]- | 159.102 | 159.1026 | -0.2 | HMDB0013899 | ↓ |
| 45 | 1.97 | Thymidine | C10H14N2O5 | [M-H]- | 241.082 | 241.0828 | -0.9 | HMDB0000273 | - |
| 46 | 8.97 | LysoPE(20:5(5Z,8Z,11Z,14Z,17Z)/0:0) | C25H42NO7P | [M-H]- | 498.2625 | 498.2628 | 0.4 | HMDB0011519 | ↓ |
| 47 | 18.11 | PE(18:0/20:5(5Z,8Z,11Z,14Z,17Z)) | C43H76NO8P | [M+HCOO] | 824.55 | 824.5472 | 3 | HMDB0009005 | ↓ |
| 48 | 17.55 | PE(20:0/18:2(9Z,12Z)) | C43H82NO8P | [M+HCOO] | 830.595 | 830.5944 | 3.3 | HMDB0009225 | ↓ |
| 49 | 20.13 | PE(18:2(9Z,12Z)/20:1(11Z)) | C43H80NO8P | [M+HCOO] | 828.5709 | 828.5766 | 0.7 | HMDB0009098 | ↓ |
| 50 | 19.54 | PE(18:0/22:6(4Z,7Z,10Z,13Z,16Z,19Z)) | C45H78NO8P | [M+HCOO] | 850.5652 | 850.5632 | 3.4 | HMDB0009012 | - |

**Table S3.** Basic information of bioactive compounds of Gynura procumbens

| No | Molecule ID | Molecule Name | Formula | MW | AlogP | Hdon | Hacc | OB% | DL | Drug-likeness Weight | Structure |
| --- | --- | --- | --- | --- | --- | --- | --- | --- | --- | --- | --- |
| GP1 | MOL002237 | 3-Carboxy-4-hydroxy-phenoxy glucoside | C13H16O9 | 316.26074 | -0.954 | 6 | 9 | 13.85 | 0.22 | 0.412 | 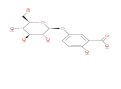 |
| GP2 | MOL000103 | 4-Hydroxybenzoic acid | C7H6O4 | 138.13 | 1.17 | 2 | 3 | 30.15 | 0.03 | 0.539 | 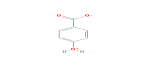 |
| GP3 | MOL003319 | 4-Hydroxyphenylacetic acid | C8H8O3 | 151.15 | 0.52 | 1 | 3 | 41.89 | 0.03 |  | 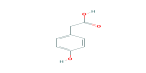 |
| GP4 | MOL000432 | a-Linolenic acid | C18H30O2 | 278.4296 | 5.971 | 1 | 2 | 45.01 | 0.15 | 0.421 | 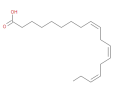 |
| GP5 | MOL001314 | Azelaic acid | C9H16O4 | 188.22093 | 1.921 | 2 | 4 | 16.90 | 0.04 | 0.61 | 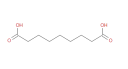 |
| GP6 | MOL000219 | Benzoic acid | C7H6O2 | 121.12 | 0.76 | 0 | 2 | 31.55 | 0.02 | 0.612 | 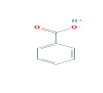 |
| GP7 | MOL000414 | Caffeic acid | C9H8O4 | 180.17 | 1.37 | 3 | 4 | 54.97 | 0.05 | 0.475 | 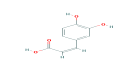 |
| GP8 | MOL001456 | Citric acid | C6H8O7 | 192.14 | -1.39 | 4 | 7 | 56.22 | 0.05 | 0.397 | 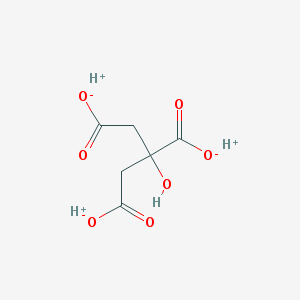 |
| GP9 | MOL000676 | Dibutyl phthalate | C16H22O4 | 278.38 | 4.2 | 0 | 4 | 64.54 | 0.13 | 0.602 | 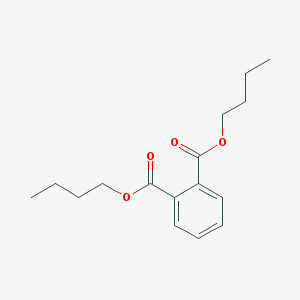 |
| GP10 | MOL004368 | Hyperoside | C16H12O6 | 300.26287 | 2.614 | 4 | 6 | 60.94 | 0.77 | 0.603 | 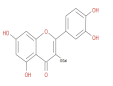 |
| GP11 | MOL001737 | Indole-3-carboxylic acid | C9H7NO2 | 161.17 | 1.73 | 2 | 2 | 33.86 | 0.05 |  | 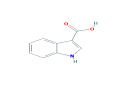 |
| GP12 | MOL000422 | Kaempferol | C15H10O6 | 286.25 | 1.77 | 4 | 6 | 41.88 | 0.24 | 0.637 | 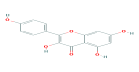 |
| GP13 | MOL000131 | Linoleic acid | C18H32O2 | 280.5 | 6.39 | 1 | 2 | 41.9 | 0.14 | 0.294 | 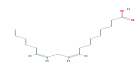 |
| GP14 | MOL000006 | Luteolin | C15H10O6 | 286.25 | 2.07 | 4 | 6 | 36.16 | 0.25 | 0.598 | 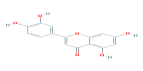 |
| GP15 | MOL001393 | Myristic acid | C14H28O2 | 228.37091 | 5.48 | 1 | 2 | 21.18 | 0.07 | 0.48 | 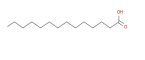 |
| GP16 | MOL001739 | Palmitoleic acid | C16H30O2 | 254.46 | 5.92 | 1 | 2 | 35.78 | 0.1 | 0.412 | 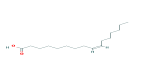 |
| GP17 |  | Phaseoloidin | C14H18O9 | 330.28732 | -0.92 | 6 | 9 |  |  | 0.402 | 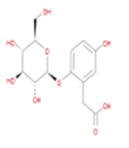 |
| GP18 | MOL000771 | p-Hydroxycinnamic acid | C9H8O3 | 164.17 | 1.64 | 2 | 3 | 43.29 | 0.04 | 0.654 | 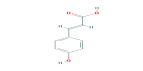 |
| GP19 | MOL000098 | Quercetin | C15H10O7 | 302.25 | 1.5 | 5 | 7 | 46.43 | 0.28 | 0.506 | 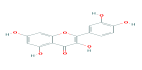 |
| GP20 | MOL004544 | Quinic acid | C7H12O6 | 192.19 | -2.39 | 5 | 6 | 63.53 | 0.06 | 0.317 | 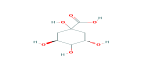 |
| GP21 | MOL000415 | Rutinum | C17H14O6 | 314.28945 | 3.071 | 4 | 6 | 32.23 | 0.68 | 0.635 | 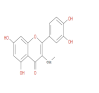 |
| GP22 | MOL001801 | salicylic acid | C7H6O3 | 138.12073 | 1.217 | 2 | 3 | 32.13 | 0.03 | 0.613 | 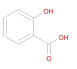 |
| GP23 | MOL002152 | Sinapic acid | C11H12O5 | 224.23 | 1.6 | 2 | 5 | 64.15 | 0.08 | 0.76 | 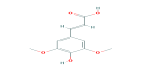 |
| GP24 | MOL002083 | Tricin | C17H14O7 | 330.28886 | 2.377 | 3 | 7 | 27.86 | 0.34 | 0.793 | 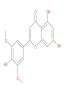 |
| GP25 | MOL000114 | Vanillic acid | C8H8O4 | 168.16 | 1.15 | 2 | 4 | 35.47 | 0.04 | 0.696 | 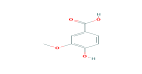 |

**Table S4.** Hub genes of treating type 2 diabetic of Gynura procumbens Prescription and its topological properties

| Gene Name | Uniprot ID | Target | Target class | Degree |
| --- | --- | --- | --- | --- |
| INS | P01308 | Insulin | insulin/IGF/relaxin family | 83 |
| AKT1 | P31749 | AKT serine/threonine kinase1 | Calcium-binding protein; kinase; transfer/carrier protein; transferase | 77 |
| MAPK1 | P28482 | Mitogen-activated protein kinase 1 | Kinase; transferase | 66 |
| CASP3 | P42574 | Caspase-3 | Peptidase | 65 |
| NOS3 | P29474 | Nitric oxide synthase, endothelial | Nitric oxide synthase | 54 |
| CASP8 | Q14790 | Caspase-8 | Peptidase_C14 | 45 |
